# Caregiver Feeding Practices and Late-Preschool BMI-for-Age z-Score Trajectories Among WIC-Enrolled Children: A National Longitudinal Study

**DOI:** 10.3390/nu18142249

**Published:** 2026-07-09

**Authors:** Qutaibah Oudat, Sarah E. Messiah, Stephanie Pitts, Alia Ghoneum

**Affiliations:** 1 Department of Biobehavioral Health & Nursing Science, College of Nursing, University of South Carolina, Columbia, SC 29208, USA; 2 Department of Epidemiology, Peter O’Donnell Jr. School of Public Health, University of Texas Southwestern Medical Center, Dallas, TX 75390, USA; sarah.messiah@utsouthwestern.edu; 3 Department of Public Health, East Carolina University, Greenville, NC 27858, USA; jilcotts@ecu.edu; 4 Department of Family Medicine, East Carolina University, Greenville, NC 27858, USA; ghoneuma23@ecu.edu

**Keywords:** childhood obesity, BMI-for-age z-score, WIC, preschool, caregiver feeding practices, BMIz trajectories, longitudinal cohort, health disparities

## Abstract

Background/Objectives: Early childhood is an important period when growth and adiposity patterns emerge and may persist into later childhood. Children enrolled in the Special Supplemental Nutrition Program for Women, Infants, and Children (WIC) face elevated obesity risk. We characterized BMI-for-age z-score (BMIz) trajectories from 24 to 60 months and their child-, caregiver-, and household-level correlates. Methods: This was a secondary analysis of the WIC Infant and Toddler Feeding Practices Study-2 (ITFPS-2) public-use dataset. We analyzed 5,583 BMIz observations from 2,247 dyads; adjusted models used 4,314 observations from 1,738 children. Linear mixed-effects models tested individual, interpersonal, and household predictors. Restriction and pressure-to-eat were lagged, time-varying exposures. Results: Mean BMIz was elevated at all waves (0.46 to 0.56). Obesity prevalence rose from 15.4% to 18.5%. Unconditionally, BMIz was stable from 24 to 48 months and rose by 60 months. Adjusted models linked higher BMIz to higher birth weight, Hispanic or Latino ethnicity, birth complications, maternal overweight or obesity, paternal overweight, prenatal smoking, adolescent maternal age, and lower maternal education. Male sex was associated with lower BMIz. Prior-wave restriction was associated with higher, and pressure to eat with modestly lower, subsequent BMIz. Household indicators showed no independent association. Conclusions: Caregiver and perinatal characteristics and feeding practices were the most consistent modifiable correlates of late-preschool BMIz. These observational, complete-case findings warrant caution. The 48–60-month window may be a promising period to target for WIC-based obesity prevention.

## 1. Introduction

Early childhood is a period of rapid growth, and BMI patterns begin to emerge during these years. Prospective cohort studies show that early biological, behavioral, and caregiving exposures are associated with distinct BMI z-score trajectories and obesogenic growth patterns across the first 6 years of life [1]. In particular, higher or rapidly increasing BMI-for-age z-score (BMIz) trajectories are associated with a greater likelihood of persistent obesity [2,3,4]. Moreover, higher birth weight, rapid early weight gain, and accelerated BMI increase in infancy are established predictors of later adiposity and metabolic risk [5,6,7].

In the USA, obesity among children aged 2–5 years remains a persistent public health challenge. Notably, it shows marked socioeconomic and racial or ethnic disparities [8,9]. In particular, children from low-income households experience disproportionately higher obesity risk. To address such risks, the Special Supplemental Nutrition Program for Women, Infants, and Children (WIC) serves more than half of U.S. infants and young children. As a result, WIC represents a major federal platform for early-life obesity prevention through provision of supplemental foods, breastfeeding support, and nutrition education [10]. Although WIC participation is associated with improved dietary quality and feeding practices, obesity prevalence among WIC-enrolled children remains high. This persistence suggests that additional biological and caregiving determinants shape early growth in this population [11,12]. To date, existing WIC-based research has focused on infant feeding, breastfeeding, and early dietary quality [13,14]. By contrast, few studies have examined how child-, caregiver-, and household-level factors relate to BMI development across the preschool period in WIC-enrolled populations. Large longitudinal cohorts indicate that parental adiposity, prenatal smoking exposure, caregiver sociodemographic characteristics, and feeding practices influence early BMI trajectories [3,15,16]. However, these cohorts underrepresent families experiencing sustained economic vulnerability and the policy-relevant context of nutrition assistance program participation. Despite this evidence, BMIz trajectories across the preschool period and their multilevel determinants remain poorly characterized among WIC-enrolled children. The present study extends prior ITFPS-2 and WIC-based analyses in two ways. First, it describes BMIz patterns from 24 to 60 months in a nationally derived WIC cohort. Second, it examines child-, caregiver-, and household-level correlates, including lagged feeding practices, within a single multilevel framework.

**Figure 1:**
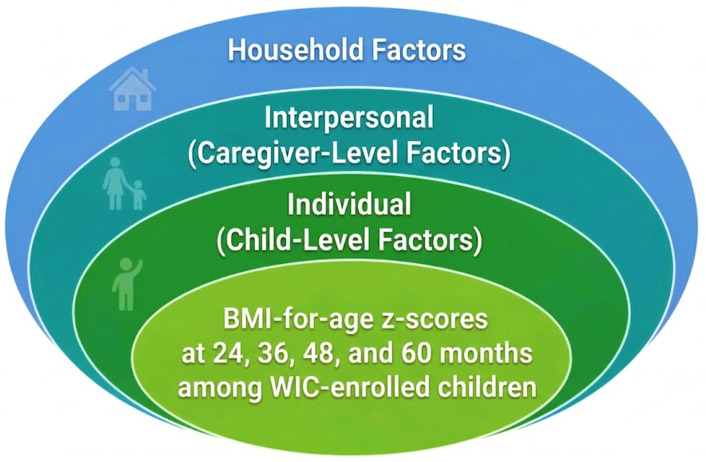
Conceptual model of multilevel determinants of BMI-for-age z-score trajectories in early childhood, guided by the Social Ecological Model (SEM).

To address this gap, the present study was guided by a conceptual model derived from the Social Ecological Model (SEM; Figure 1) [17]. This model emphasizes that distal contextual conditions, such as household socioeconomic status and food security, may influence child adiposity primarily through more proximal child and caregiver processes, including maternal health, prenatal exposures, and caregiving and feeding practices. Specifically, the study examined longitudinal associations between child-, caregiver-, and household-level factors, including lagged caregiver feeding practices, and BMI-for-age z-scores at 24, 36, 48, and 60 months among WIC-enrolled children. We hypothesized that: (1) higher birth weight, birth complications, maternal overweight or obesity, and prenatal smoking exposure would be associated with higher BMIz across early childhood; (2) greater use of restrictive and pressuring feeding practices would be associated with higher subsequent BMIz, whereas responsive feeding practices would be associated with lower subsequent BMIz; and (3) household socioeconomic and food security indicators would show limited independent associations with BMIz after accounting for child- and caregiver-level factors.

## 2. Methods

### 2.1. Study Design and Data Source

This study was a secondary analysis of the WIC Infant and Toddler Feeding Practices Study–2 (ITFPS-2). ITFPS-2 is a nationally derived, longitudinal cohort of caregiver–child dyads enrolled in the Special Supplemental Nutrition Program for Women, Infants, and Children (WIC). WIC program eligibility criteria and ITFPS-2 study-specific inclusion criteria are described in Supplementary Note S1. The cohort followed participants from infancy through early childhood to characterize feeding practices, nutrition behaviors, and growth [10,18,19]. For the present analysis, the focus was on child BMI-for-age z-scores (BMIz) assessed at 24, 36, 48, and 60 months. Data were collected through three sources: repeated caregiver interviews, WIC administrative records, and standardized child anthropometry. The study is reported in accordance with the Strengthening the Reporting of Observational Studies in Epidemiology (STROBE) guidelines [20]. The ITFPS-2 public-use data are fully de-identified; therefore, this secondary analysis did not constitute human subjects research.

### 2.2. Study Population, Sample Size, and Data Collection

ITFPS-2 enrolled caregiver–infant dyads between July 2013 and January 2014 and followed them through the child’s fifth birthday. The publicly available ITFPS-2 analytic files include child- and caregiver-level data organized by age intervals spanning birth through 60 months. Detailed descriptions of sampling, weighting, and data collection procedures have been published previously [10,18,21].

### 2.3. Eligibility Criteria and Analytic Sample

**Figure 2:**
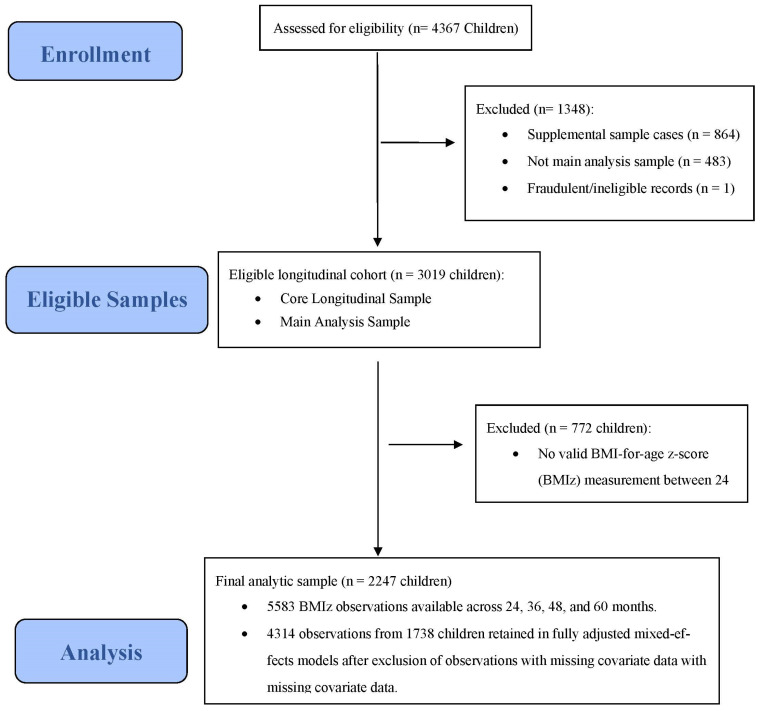
Sample selection flowchart for the longitudinal analysis of BMI-for-age z-score trajectories among WIC-enrolled children.

Children were eligible for inclusion if they met two criteria: (1) participation in the ITFPS-2 public-use longitudinal cohort and (2) at least one valid BMI-for-age z-score (BMIz) measurement between 24 and 60 months. Consistent with ITFPS-2 documentation, two groups were excluded prior to analysis: records flagged as invalid and dyads enrolled exclusively in supplemental samples. After these exclusions, the analytic sample comprised 2,247 children contributing 5,583 BMIz observations across the 24-, 36-, 48-, and 60-month assessments. Finally, after excluding observations with missing covariate data, 4,314 observations from 1,738 children were retained in the fully adjusted mixed-effects models (Figure 2). Children excluded from these models due to missing covariate data contributed fewer BMIz assessments. On average, they had more socioeconomically vulnerable profiles than those retained (e.g., lower maternal education, higher poverty exposure). The complete-case sample therefore slightly under-represents the highest-risk families.

### 2.4. Measurements and Variables

#### 2.4.1. Primary Outcomes

The primary outcome was child BMIz at 24, 36, 48, and 60 months. BMIz values were derived from measured weight and length/height collected through WIC measurement procedures and were pre-calculated by Westat using established growth chart reference standards. To document which standard was applied at each wave, the ITFPS-2 public-use codebook includes a wave-specific indicator variable (Source0m, Source6m, Source12m, Source24m, Source36m, Source48m, Source60m) that records whether the CDC 2000 or WHO 2006 reference was used to derive each child’s BMIz at that wave. At the 36-, 48-, and 60-month waves, CDC 2000 charts were applied to all children (n = 1,886, 2,115, and 1,825, respectively; WHO n = 0 at each wave). This confirmed a consistent reference standard at these waves [22]. By contrast, the reference standard at the 24-month wave varied by the child’s actual age at measurement. Specifically, in the full ITFPS-2 public-use file, children measured before 24 months used WHO 2006 standards (n = 569), whereas those measured at or after 24 months used CDC 2000 standards (n = 1,162). This split reflects the convention that WHO standards apply to children under 24 months and CDC standards from 24 months onward. CDC charts begin at exactly 24 months and cannot be validly extrapolated to younger ages. As a result, mixing standards within the same wave produced a systematic discontinuity: WHO-derived BMIz was 0.75 units higher on average than CDC-derived BMIz at this wave (1.21 vs. 0.46). This inflation of the wave-1 mean produced an artifactual decline from 24 to 36 months. Accordingly, in the analytic cohort used for trajectory modeling, to maintain a consistent CDC reference across all four waves, the 24-month BMIz was restricted to children measured at ≥24 months (n = 998). In addition, eleven biologically implausible values (<−5 or >+5 SD) were excluded across all waves, per WHO and CDC guidance [22,23].

#### 2.4.2. Exposure Variables

Variables were selected a priori and assigned distinct analytic roles consistent with the SEM (Figure 1). The lagged, time-varying caregiver feeding practices (restriction and pressure to eat) were the primary exposures of interest; they are described below. The remaining variables were a priori covariates, organized into child-, caregiver-, and household-level domains. Child- and caregiver-level factors were examined both as correlates of interest in their own right and as potential confounders of the feeding-practice associations. Household socioeconomic and food-security factors were treated as distal contextual covariates. Consistent with the SEM (Figure 1), these distal factors were expected to influence BMIz primarily through more proximal child and caregiver processes. Child-level factors included sex, race, ethnicity, birth weight (kg), and birth complications. Caregiver-level factors included maternal age at childbirth, marital status, educational attainment, parity, maternal BMI category at screening, prenatal smoking, postpartum depressive symptoms (Edinburgh Postnatal Depression Scale [EPDS] ≥10), paternal presence in the household, and paternal weight status. By contrast, prenatal alcohol use was not included as a covariate. Its prevalence in this WIC-enrolled sample was very low (1.2%), which precluded stable model estimation. Household-level factors included food security status, participation in non-WIC benefit programs, timing of WIC enrollment, and household income relative to federal poverty thresholds. Several covariates warrant additional definition. Birth complications were a binary indicator. They were coded from ITFPS-2 items documenting any perinatal complication (e.g., preterm birth, low Apgar score, neonatal intensive care unit admission, or other clinician-reported complications). Maternal BMI category at screening reflected weight status at WIC enrollment (underweight/normal weight, overweight, or obesity). It was based on height and weight recorded at the initial WIC visit, which typically occurs in early pregnancy or the postpartum period rather than strictly pre-pregnancy. Prenatal smoking was defined as the self-reported number of cigarettes smoked per day during pregnancy, reported at the baseline interview. Paternal weight status was based on caregiver report of the biological father’s BMI category.

Caregiver feeding practices were assessed at child ages 15, 42, and 54 months. Items were adapted from the Comprehensive Feeding Practices Questionnaire (CFPQ) [24], rated on a 5-point Likert scale (“never” to “always”), and reverse-coded as appropriate. Subscale scores were the mean of available items, with higher scores indicating greater use of each practice. Because feeding practices were not measured at every BMIz wave, two domains, pressure to eat and restriction, were modeled as lagged, time-varying primary exposures, such that earlier measurements preceded the subsequent BMIz outcomes. Consequently, lagged scores were available at every outcome wave from 24 to 60 months. In the present sample, both feeding subscales were reasonably stable across the 15-, 42-, and 54-month waves. Overall stability coefficients were α = 0.70 for pressure to eat and α = 0.76 for restriction. Moreover, average inter-wave correlations were 0.44 and 0.51, respectively. Stability was weakest at the 15-month wave for both subscales (corrected item-total r = 0.43 and 0.51, respectively), consistent with developmental shifts in feeding dynamics between toddlerhood and the preschool period. The ITFPS-2 feeding items were adapted from the original CFPQ for telephone administration in the WIC context, while preserving the underlying subscale constructs [24]. In the present analysis, we focused on pressure to eat and restriction for three reasons. These subscales capture controlling feeding practices with the most consistent links to child BMIz in prior work. They were available at all three feeding waves. They also showed adequate stability in this sample. Other CFPQ-derived domains (e.g., monitoring, teaching about nutrition, modeling, and encouraging variety) were available but were not modeled as primary exposures.

### 2.5. Statistical Analysis Plan and Assumption Checks

All analyses were conducted in Stata (StataCorp LLC, College Station, TX, USA; version 18) [25]. Data were structured in long format, with one record per child per assessment age. All models were estimated without survey weights because the ITFPS-2 public-use analytic file does not include sampling or attrition weights or clustering and stratification identifiers; design-based adjustment was therefore not possible. Descriptive characteristics were summarized using means (standard deviations) for continuous variables and frequencies (percentages) for categorical variables. Change in BMI-for-age z-score (BMIz) from 24 to 60 months and its correlates were examined using linear mixed-effects models. Each model included a child-specific random intercept to account for within-child correlation across repeated measurements. In addition, child age was modeled as a categorical fixed effect (24, 36, 48, and 60 months; reference = 24 months) to allow for non-linear BMIz change during early childhood. These models used child-specific random intercepts and modeled age as a categorical fixed effect. They therefore estimate population-averaged BMIz differences across age waves and covariate groups. They do not estimate individual growth trajectories or distinct trajectory classes.

An unconditional model including only child age was fit first to characterize mean BMIz from 24 to 60 months and to estimate the intraclass correlation coefficient (ICC). A fully adjusted model then added four blocks of covariates. Guided by the Social Ecological Model, these blocks were specified to represent individual (child), interpersonal (caregiver and feeding practices), and household (socioeconomic) levels of influence on BMIz. This SEM framework informed the grouping and ordering of predictors, while all variables were estimated jointly in a single fully adjusted model. First, child-level factors comprised birth weight, sex, race, ethnicity, and birth complications. Second, caregiver-level factors comprised maternal age at childbirth, marital status, parity, maternal weight status, prenatal smoking, education, postpartum depressive symptoms, paternal presence, and paternal weight status. Third, household-level factors comprised food security, other benefit program participation, timing of WIC enrollment, and household poverty level. Finally, lagged time-varying feeding practices (pressure to eat and restriction) were entered as the primary exposures. To preserve temporal ordering, feeding practices were modeled as lagged predictors, with practices measured at 15 months entered as predictors of BMIz at 24 and 36 months, practices measured at 42 months as predictors of BMIz at 48 months, and practices measured at 54 months as predictors of BMIz at 60 months. A prespecified sensitivity analysis re-estimated the fully adjusted model without birth weight. This step was taken because birth weight may play a dual role as both confounder and mediator between prenatal exposures and early adiposity (Supplementary Table S1).

Extreme BMI-for-age z-score values (<−5 or >+5 SD) were considered biologically implausible. This threshold followed World Health Organization and Centers for Disease Control and Prevention guidance. Accordingly, eleven BMIz observations were set to missing prior to analysis and excluded on this basis. All mixed-effects models were estimated using restricted maximum likelihood (REML). Model assumptions were then assessed graphically: residual-versus-fitted plots were inspected for nonlinearity and heteroscedasticity, and Q–Q plots were used to assess normality of level-1 residuals and random effects. Influential clusters were assessed by refitting models after excluding the 1% of children with the largest absolute random-intercept BLUPs. Results were materially unchanged. In addition, variance inflation factors for all predictors, including feeding practices, were <1.2, indicating negligible collinearity. Covariates with insufficient prevalence for stable estimation (prenatal alcohol use) were excluded from multivariable models. By contrast, all remaining covariates were retained in the fully adjusted model for theoretical completeness.

## 3. Results

### 3.1. Participant Characteristics

Table 1 presents baseline characteristics of the analytic sample. In total, the analytic cohort comprised 2,247 WIC-enrolled caregiver–child dyads. Together, these dyads contributed 5,583 BMI-for-age z-score (BMIz) observations across the 24-, 36-, 48-, and 60-month assessments. After restricting 24-month BMIz to CDC-derived observations and excluding biologically implausible values, per-wave counts were n = 998, 1,503, 1,654, and 1,428 at 24, 36, 48, and 60 months, respectively. Overall, the mean BMIz across all available observations was 0.51 ± 1.29. On average, children contributed between one and four BMIz assessments across follow-up.

At the child level, approximately half of children were female (48.7%), and 45.6% were Hispanic or Latino. By race, most children were identified as White (58.6%), followed by Black or African American (23.8%) and other racial groups (17.6%). Mean birth weight was 3.23 ± 0.50 kg. In addition, 11.5% of children experienced birth complications. At the caregiver level, nearly half of mothers were aged ≥26 years (48.9%) at baseline, and 40.7% were aged 20–25 years. Most caregivers were not married (68.8%), and 41.1% of children were first-born. With respect to maternal weight status, 29.8% of mothers had obesity at screening and 24.6% were overweight. Daily cigarette smoking during pregnancy was reported by 10.7% of caregivers. By contrast, prenatal alcohol use was uncommon (1.2%) and was therefore not included in multivariable models due to insufficient prevalence for stable estimation. With regard to education, more than half of caregivers had a high school education or less (61.3%). At the household level, socioeconomic vulnerability was prevalent. Specifically, nearly half of households experienced low or very low food security (48.8%), and 62.8% were living at or below 75% of the federal poverty guideline. In addition, 49.3% of families participated in SNAP, alone or in combination with other programs. Finally, most caregivers enrolled in WIC during the first or second trimester of pregnancy (73.4%).

Descriptive statistics for caregiver feeding practices in early childhood are shown in Table 2. First, mean pressure-to-eat scores rose modestly across the preschool period (2.97 ± 1.13 at 15 months, n = 1,699; 3.18 ± 1.10 at 42 months, n = 1,892; 3.23 ± 1.09 at 54 months, n = 1,854). By contrast, mean restriction scores declined over the same period (3.53 ± 1.37, 3.43 ± 1.39, and 3.37 ± 1.37 at 15, 42, and 54 months, respectively). Overall, all scores ranged from 1 to 5, the full instrument scale.

### 3.2. BMI-for-Age z-Score Patterns Across Developmental Stages

The analytic sample for the trajectory models included 5,583 BMI-for-age z-score (BMIz) observations from 2,247 children (mean observations per child = 2.5; range = 1–4). On average, the mean BMIz was modestly above zero at all waves, indicating a slight upward shift relative to the reference population (24 months: 0.46, SD 1.25; 36 months: 0.50, SD 1.27; 48 months: 0.50, SD 1.34; 60 months: 0.56, SD 1.29; Table 1). In parallel, the proportion of children meeting the threshold for overweight or obesity (BMIz ≥ 1.036) ranged from 31.1% at 36 months to 35.1% at 60 months. Similarly, the proportion meeting the threshold for obesity (BMIz ≥ 1.645) increased from 15.4% at 24 months to 18.5% at 60 months. Overall, these patterns suggest a gradual upward trend in adiposity risk across early childhood.

As summarized in Table 3, an unconditional two-level mixed-effects model with a random intercept for child was estimated using restricted maximum likelihood. This model was used to characterize the BMIz trajectory and to partition variance. The intraclass correlation coefficient (ICC) was 0.63 (95% CI 0.61–0.65). Specifically, 63% of the total BMIz variance reflected stable between-child differences, and 37% reflected within-child variation. Accordingly, this finding justified multilevel modeling for all subsequent analyses.

**Figure 3:**
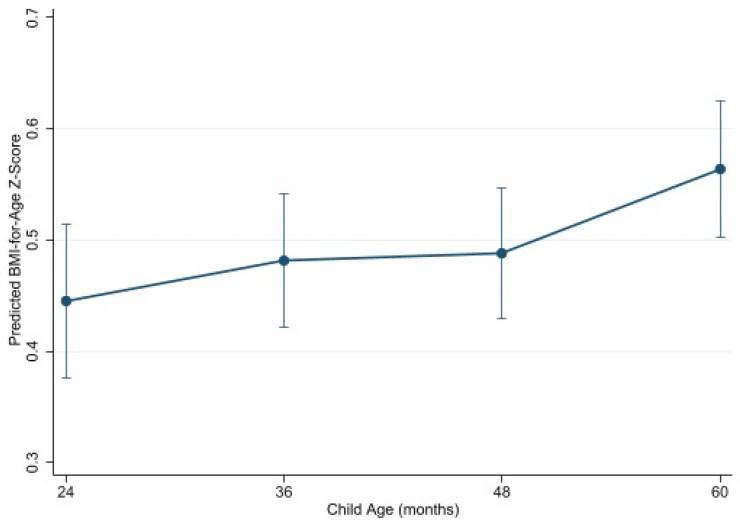
Unconditional mixed-effects model estimates of predicted BMI-for-age z-score (BMIz) trajectories from 24 to 60 months.

In the unconditional model, the mean BMIz at 24 months was 0.45 (95% CI 0.38–0.51; *p* < 0.001). Values at 36 and 48 months did not differ significantly from 24 months (β = 0.04; *p* = 0.307 and 0.219, respectively). However, BMIz rose by 60 months (β = 0.12; SE = 0.04; 95% CI 0.05–0.19; *p* = 0.001; estimated marginal mean 0.56, 95% CI 0.50–0.62). Overall, BMIz remained stable from 24 to 48 months and then shifted modestly upward by 60 months (Wald χ^2^(3) = 13.07, *p* = 0.005; Figure 3).

### 3.3. Factors Associated with BMI-for-Age z-Scores Across Early Childhood

Associations reported in Section 3.3.1, Section 3.3.2 and Section 3.3.3 are drawn from the fully adjusted model (Table 4; 4,314 observations from 1,738 children). This model included child-, caregiver-, and household-level covariates alongside lagged, time-varying feeding practices. Between-child heterogeneity remained substantial (ICC = 0.58, 95% CI 0.55–0.61). This estimate represents a modest reduction from the unconditional ICC of 0.63. Accordingly, the predictors included explained only a small share of between-child variability. Finally, a likelihood-ratio test confirmed the need for the mixed-effects structure (*p* < 0.001).

#### 3.3.1. Individual (Child-Level Factors)

Several child-level characteristics were independently associated with BMIz across early childhood. First, higher birth weight was strongly associated with higher BMIz (β = 0.51, SE = 0.06, *p* < 0.001, 95% CI [0.40, 0.62]). Second, male children had significantly lower BMIz than female children (β = −0.16, SE = 0.05, *p* = 0.004, 95% CI [−0.27, −0.06]). In addition, children of Hispanic or Latino ethnicity had higher BMIz relative to non-Hispanic children (β = 0.19, SE = 0.06, *p* = 0.005, 95% CI [0.07, 0.31]). Similarly, children who experienced birth complications had higher BMIz than those without complications (β = 0.25, SE = 0.09, *p* = 0.004, 95% CI [0.08, 0.42]). By contrast, race was not significantly associated with BMIz after adjustment.

#### 3.3.2. Interpersonal (Caregiver-Level Factors)

Several caregiver characteristics were independently associated with child BMIz. First, maternal obesity at screening was the strongest caregiver-level predictor (β = 0.41, SE = 0.06, 95% CI 0.28–0.53; *p* < 0.001), followed by maternal overweight (β = 0.16, SE = 0.07, 95% CI 0.03–0.30; *p* = 0.014). Second, daily prenatal smoking showed a dose-dependent positive association (1–9 cigarettes/day: β = 0.47, SE = 0.10, 95% CI 0.27–0.67; *p* < 0.001; 10–20 cigarettes/day: β = 0.49, SE = 0.16, 95% CI 0.17–0.82; *p* = 0.003). By contrast, maternal education above high school was associated with lower child BMIz (β = −0.13, SE = 0.06, 95% CI −0.25 to −0.02; *p* = 0.021). With respect to maternal age, children of mothers aged 20–25 years had lower BMIz than those of the youngest mothers (16–19 years; β = −0.24, SE = 0.10, 95% CI −0.44 to −0.04; *p* = 0.018), whereas the ≥26-year group showed the same direction but did not reach significance. Baseline biological father overweight (vs. underweight) was also associated with higher child BMIz (β = 0.35, SE = 0.15, 95% CI 0.06–0.65; *p* < 0.05). By contrast, maternal marital status was not significantly associated with BMIz after full adjustment. Turning to feeding practices, greater lagged restriction was associated with higher subsequent BMIz (β = 0.07, SE = 0.01, 95% CI 0.04–0.10; *p* < 0.001), whereas greater lagged pressure to eat was associated with lower subsequent BMIz (β = −0.04, SE = 0.02, 95% CI −0.08 to −0.01; *p* = 0.013). Notably, both feeding-practice associations persisted after mutual adjustment and full covariate control.

#### 3.3.3. Household Factors

No household-level indicator was independently associated with BMIz in the fully adjusted mixed-effects model (Supplementary Table S1). Poverty level showed no association (75–130% FPL: β = −0.01, *p* = 0.897; >130% FPL: β = −0.01, *p* = 0.903; ref. ≤75% FPL). Food security likewise was not associated (low: β = 0.01, *p* = 0.825; very low: β = 0.01, *p* = 0.908; ref. high/marginal). Benefit-program participation showed null associations (SNAP or SNAP + other: β = −0.13, *p* = 0.133; other non-SNAP: β = −0.01, *p* = 0.922; ref. none). Finally, timing of WIC enrollment was not associated with BMIz (2nd trimester: β = 0.03, *p* = 0.581; 3rd trimester: β = 0.05, *p* = 0.540; postnatal: β = −0.02, *p* = 0.869; ref. 1st trimester).

### 3.4. Sensitivity Analyses

A prespecified sensitivity analysis excluded birth weight from the fully adjusted mixed-effects model to assess whether feeding-practice associations depended on this perinatal covariate. Excluding birth weight slightly reduced overall model fit but left the magnitude and direction of feeding-practice coefficients essentially unchanged. In this sensitivity model, lagged pressure to eat remained negatively associated with BMIz (β = −0.04, *p* = 0.013, 95% CI [−0.08, −0.01]), and lagged restriction remained positively associated (β = 0.07, *p* < 0.001, 95% CI [0.04, 0.10]). These findings indicate that caregiver feeding-practice associations with child BMIz are robust to whether birth weight is included in the model (Supplementary Table S1).

## 4. Discussion

In this nationally derived cohort of WIC-enrolled children, BMIz remained stable from 24 to 48 months, with a modest upward shift by 60 months. Accordingly, continued BMIz monitoring through the preschool period appears warranted. At the child level, higher birth weight, Hispanic or Latino ethnicity, and birth complications were associated with higher BMIz, whereas male sex was associated with lower BMIz. At the caregiver level, maternal overweight and obesity, prenatal smoking, and lower educational attainment were associated with higher child BMIz, and baseline biological father overweight (vs. underweight) was likewise positively associated with BMIz. In addition, lagged feeding practices showed opposing directions: greater restriction was associated with higher subsequent BMIz, whereas greater pressure to eat was associated with modestly lower BMIz. By contrast, household factors, including poverty level, food security, benefit-program participation, and timing of WIC enrollment, were not independently associated with BMIz after full adjustment. Taken together, these findings highlight the central role of caregiver characteristics, paternal weight status, and feeding contexts. Importantly, the observed associations are correlational and do not imply causation.

### 4.1. BMIz Trajectories Across Early Childhood

In the fully adjusted model (Table 4), mean BMIz did not differ significantly from 24 months at 36 months. It then rose modestly by 48 months and increased further by 60 months (48 months: β ≈ 0.10, *p* = 0.024; 60 months: β ≈ 0.18, *p* < 0.001). By contrast, the unconditional model showed no significant differences at 36 or 48 months (Section 3.2). This pattern suggests that covariate adjustment sharpens the timing of the upward shift in BMIz. Moreover, these population-averaged differences resemble the gradual rise in BMI that typically follows infancy during the preschool years [4]. However, this study assessed BMIz at four discrete ages rather than identifying individual BMI nadirs. It therefore does not directly estimate adiposity rebound. The pattern is better described as a modest late-preschool upward shift in BMIz. Accordingly, the mixed-effects models describe mean BMIz patterns across the 24-, 36-, 48-, and 60-month waves and their associations with predictors. They do not capture heterogeneous within-child trajectory shapes or age-by-predictor interactions.

The unconditional ICC of 0.63 indicates that roughly two-thirds of BMIz variance reflected stable between-child differences; the fully adjusted ICC of 0.58 shows that the included predictors explained only a small share of that between-child variability. The models therefore primarily characterize which children carry higher average BMIz, not how BMIz changes within individuals.

Although modest in magnitude, the significant increase at 60 months was consistent across models. This finding indicates an emerging upward shift in population-level adiposity during the late preschool period. Similar patterns have been reported in the ECHO study and other trajectory-based analyses, where most children exhibit BMI stability, and only a minority show early acceleration [3,26,27]. Comparable patterns also appear in European preschool cohorts between approximately 3 and 6 years, particularly among children with more favorable movement behaviors [28]. Notably, even in a nutritionally vulnerable WIC population, pronounced divergence in BMIz may not yet be evident in the early preschool years. Because earlier or steeper increases in BMI during this period are associated with greater obesity risk later [4], the 48-to-60-month shift may represent a transitional window for early prevention within WIC [3].

### 4.2. Individual (Child-Level Factors)

Consistent with existing evidence [29,30,31,32,33,34,35], higher birth weight was strongly associated with elevated BMIz across early childhood. Population-based and meta-analytic evidence links higher birth weight and large-for-gestational-age status to greater BMIz and elevated overweight risk later in childhood. For example, children born >4000 g have nearly doubled odds of overweight compared with normal-birth-weight peers, independent of sociodemographic and perinatal factors [29,30,31]. These associations likely reflect fetal metabolic programming. In this pathway, in utero overnutrition and excessive gestational weight gain promote greater adiposity at birth and accelerated postnatal weight gain [35]. Birth weight is an easily identifiable early-life marker that could inform risk stratification and the timing of intensified obesity prevention in high-risk populations such as WIC.

Second, male children had modestly lower BMIz than female children. This finding suggests that early-life adiposity trajectories may differ by sex even within low-income populations. Longitudinal evidence from low-income Hispanic children aged 2–5 years shows sex-stratified patterns in BMIz and related adiposity measures [36]. Moreover, contextual influences may further modify these differences. For example, sports-focused early childhood education and care settings appear to confer greater BMI benefit among boys than girls [37]. By contrast, other preschool studies have reported higher odds of overweight and obesity among males [38]. Together, these findings underscore that sex differences are heterogeneous and context-dependent. These findings support sex-sensitive prevention strategies that consider differential activity patterns, caregiver practices, and environmental exposures.

Third, Hispanic or Latino ethnicity was associated with higher BMIz relative to non-Hispanic children. This finding is consistent with extensive U.S. surveillance and cohort evidence documenting persistent racial and ethnic disparities in childhood obesity. For instance, nationally representative NHANES analyses demonstrate that Hispanic children have a significantly higher obesity prevalence than non-Hispanic White children across multiple age groups, with no evidence of sustained decline in recent years [39,40]. In addition, longitudinal data show that Latino children exhibit higher predicted mean BMI and less favorable BMI growth trajectories during early childhood compared with White peers, even after accounting for child, maternal, and household characteristics [41]. Importantly, emerging evidence indicates substantial heterogeneity within Latino populations; for example, U.S.-born Latino children have higher odds of obesity than both foreign-born Latino and non-Hispanic White children, highlighting the potential influence of acculturation, food environments, and structural exposures on obesity risk [42]. Large pooled cohort analyses similarly report higher average BMIz among Hispanic children relative to non-Hispanic White children in several U.S. regions [43]. Overall, these findings suggest that the observed ethnic disparities in BMIz are unlikely to reflect inherent biological differences. Rather, they likely reflect differential exposure to obesogenic environments, food insecurity, neighborhood deprivation, and barriers to preventive resources. Therefore, culturally responsive and structurally informed strategies within WIC and related early childhood systems may help address these inequities. Given the observational design, this possibility should be tested in future intervention research rather than treated as an established strategy.

Finally, children with birth complications had higher BMIz. Direct longitudinal evidence linking heterogeneous neonatal complications to later adiposity is limited. Nevertheless, the association is biologically plausible. Complications often reflect substantial perinatal stress and physiologic disruption. For example, birth asphyxia signals compromised oxygenation arising from intrapartum events such as prolonged or obstructed labor [44]. In addition, complications requiring intensive neonatal support can disrupt early feeding, growth regulation, sleep–wake organization, and stress physiology [45,46]. Preterm-related morbidity likewise reflects immature organ systems and medical exposures during a sensitive developmental window, with downstream cardiometabolic vulnerability [46,47]. Therefore, birth complications are best interpreted as markers of early-life adversity. Future studies should accordingly disaggregate specific complication types and care pathways.

### 4.3. Interpersonal (Caregiver-Level Factors)

Several caregiver-level characteristics were independently associated with child BMIz. Together, these findings underscore caregiving as a proximal and influential domain after adjustment for child- and household-level factors. First, children of adolescent mothers had higher BMIz than those of young adult mothers. This pattern is consistent with evidence that adolescent childbearing functions primarily as a marker of cumulative socioeconomic disadvantage and constrained caregiving resources, rather than as a biological exposure [48,49]. Second, maternal weight status emerged as among the strongest caregiver-level correlates. Maternal overweight and obesity were associated with higher BMIz throughout the preschool years, and baseline biological father overweight (vs. underweight) was likewise positively associated with BMIz. These parental patterns are consistent with longitudinal evidence linking maternal pre-pregnancy BMI and paternal overweight to higher offspring BMI and less favorable BMI trajectories from infancy onward [50,51,52,53]. It likely reflects a combination of intrauterine programming, shared genetic susceptibility, and sustained postnatal influences [5].

Prenatal smoking was independently associated with higher child adiposity in a dose-dependent pattern, paralleling prior evidence [54,55,56,57]. Recent cohort studies using detailed body composition measures report higher BMI, fat mass index, waist circumference, and central adiposity from childhood into adulthood among prenatally exposed children, even after confounder adjustment [58]. These findings support prenatal smoking as a clinically relevant and modifiable risk factor [55,56]. In addition, children whose mothers had more than a high school education had lower BMIz. This pattern is consistent with parental education being a key social determinant of childhood adiposity. Specifically, children of lower-educated parents experience greater early weight gain and higher overweight or obesity risk across childhood and adolescence [59]. Moreover, maternal education is inversely associated with BMIz independent of maternal BMI, income, and child characteristics [60]. These pathways likely operate through health literacy, prenatal and postnatal behaviors, and the home environment.

### 4.4. Caregiver Feeding Practices (Lagged, Time-Varying)

Greater caregiver restriction at the prior assessment was associated with higher child BMIz. By contrast, greater pressure to eat was associated with modestly lower subsequent BMIz. Modeling feeding practices as lagged, time-varying exposures preserved the temporal order of measurement between practices and BMIz; however, because the intervals between assessments were short, these associations remain close to cross-sectional and are likely bidirectional. The models did not adjust for earlier BMIz. The feeding-practice coefficients therefore reflect associations with BMIz level, not effects on BMIz change. They may partly capture the child’s pre-existing size. Models that adjust for prior BMIz are a useful next step for studying change in BMIz. Controlling feeding practices are closely linked to child weight status. However, the direction of influence is complex and often bidirectional. For example, pressure to eat is more frequently directed toward children with lower BMI. In early childhood, it has been associated with lower energy intake, reduced consumption of high-fat foods, and lower BMIz [61]. Similarly, systematic reviews indicate that restriction and pressure to eat are most consistently correlated with child BMI in cross-sectional analyses. In these analyses, parents often adapt these practices in response to child weight rather than acting as primary causal drivers [62].

Longitudinal evidence supports this bidirectional framework. For instance, in adolescents, restriction and pressure to eat are cross-sectionally associated with BMIz but do not predict subsequent change. This pattern suggests that these practices more often represent parental responses than drivers of weight status [63]. Similarly, in an Asian preschool cohort, higher child BMI predicted greater subsequent maternal restriction and lower pressure to eat. However, only restriction showed a small but persistent prospective association with later BMI after adjustment for baseline BMI [64]. The 2025 Dietary Guidelines Advisory Committee systematic review likewise concluded that current evidence is insufficient and inconsistent to draw firm conclusions about controlling feeding practices and child growth or obesity risk [65]. Therefore, the present findings should be read as showing that, among WIC-enrolled families, restriction clusters with weight-related dynamics in higher-BMI children, and pressure to eat is preferentially directed toward lower-BMI children. They should not be interpreted as evidence of purely causal mechanisms. Although lagged modeling reduces some concerns about reverse causality, these associations should still be interpreted with caution. The temporal alignment between the feeding and BMIz assessments is also approximate, so the lag structure is imperfect. Residual confounding (e.g., unmeasured child appetitive traits or family stressors) and caregiver responses to children’s existing weight status remain plausible explanations. Our findings are therefore consistent with a bidirectional framework in which caregiver feeding practices both shape and respond to children’s growth, rather than providing definitive evidence of a single causal direction.

### 4.5. Household Factors

Household factors were not independently associated with child BMIz from 24 to 60 months after full multivariable adjustment. This null finding is consistent with evidence that household-level associations with early childhood BMI often attenuate after accounting for maternal characteristics, early growth, and demographic factors [66,67]. For example, in nationally representative samples, socioeconomic gradients in overweight and obesity may be weak or absent within low-income preschool populations after confounder control [68]. In addition, multiple studies report no independent association between household food insecurity and BMIz once extensive covariates are considered [67,69]. Within WIC-enrolled populations, shared program participation and limited socioeconomic variability may further constrain the detection of independent household effects. Moreover, WIC-based interventions have improved obesogenic behaviors without consistent changes in preschool BMIz [70]. Overall, these findings suggest that household and environmental conditions influence early childhood adiposity primarily through indirect pathways. Specifically, they shape caregiving constraints and behaviors rather than exerting strong direct effects on BMIz.

### 4.6. Strengths and Limitations

This study has several strengths. First, it leveraged a nationally derived longitudinal cohort of WIC-enrolled caregiver–child dyads with repeated BMIz measurements from 24 to 60 months. This design allowed for the examination of multilevel correlates of early childhood growth in a policy-relevant, high-risk population. Second, linear mixed-effects models with child-specific random intercepts appropriately accounted for within-child correlation and substantial between-child heterogeneity. Third, predictors were selected a priori across child, caregiver, and household domains. In addition, caregiver feeding practices were modeled as lagged, time-varying exposures to preserve temporal ordering. Finally, biologically implausible BMIz values were excluded using standardized rules, and model assumptions and influential clusters were systematically evaluated.

Several limitations should also be noted. First, as an observational secondary analysis, findings are associative and may reflect residual confounding. Second, geographic identifiers were unavailable. This gap precluded control for area-level factors such as food environments, healthcare access, and residential segregation that may contribute to observed ethnic disparities. Third, most measures relied on caregiver report, raising the possibility of recall and social desirability bias. Fourth, feeding practices were not measured at every wave. This limitation required lagged alignment across nonconsecutive time points and potentially introduced exposure misclassification. Because the lag intervals were short and feeding practices were measured at only a few time points, these associations are closer to cross-sectional than truly prospective and may be bidirectional. Future studies should examine whether earlier feeding practices predict later BMIz while adjusting for baseline BMIz. Fifth, missing covariate data reduced the fully adjusted sample from 2,247 to 1,738 children. Complete-case analysis may therefore bias results if missingness was not random. Furthermore, excluded children tended to have adolescent, less-educated mothers and greater socioeconomic vulnerability, indicating underrepresentation of the most disadvantaged families. In observation terms, the complete-case requirement reduced the sample from 5,583to 4,314 BMIz observations. As a result, our findings may slightly underestimate associations that are concentrated in the highest-risk families. The BMIz trajectories may also underrepresent the most disadvantaged children. We did not use multiple imputation or inverse probability weighting. Future work using these approaches would help refine inference in this cohort. Sixth, birth complications were modeled as a heterogeneous binary indicator, which limits clinical specificity. In addition, because the ITFPS-2 public-use file does not provide sampling or attrition weights or design identifiers, all analyses were unweighted. Although the cohort is nationally derived, estimates should be interpreted as describing BMIz patterns and associations within this analytic cohort rather than as fully survey-weighted national effect estimates for all WIC-enrolled children. The 24-month BMIz was restricted to children measured at ≥24 months. This avoided the WHO/CDC growth-standard discontinuity at this wave. The restriction may add minor selection effects. It also reduces comparability with studies that use all 24-month observations. Other approaches could give slightly different estimates. These include adding a growth-standard indicator, modeling exact age at measurement, or omitting the 24-month wave. We kept the CDC-only approach because it is the most transparent. In addition, child height and weight were recorded across many WIC sites under field conditions, which can introduce anthropometric measurement error. Relatedly, the adjusted models did not include prior BMIz as a covariate. The reported associations therefore reflect BMIz level rather than within-child change. Finally, generalizability is greatest to WIC-enrolled families and may not extend to more advantaged populations.

## 5. Conclusions

Among WIC-enrolled children in this national cohort, the mean BMIz was stable from 24 to 48 months. It then rose modestly at 60 months. Obesity prevalence rose from 15.4% to 18.5% over the follow-up period. Several factors were independently associated with higher BMIz: higher birth weight, birth complications, maternal overweight or obesity, prenatal smoking, lower maternal education, and paternal overweight. Household socioeconomic indicators showed no independent association after full adjustment. Two feeding practices were also linked to BMIz. Greater prior-wave restriction was associated with higher later BMIz. Greater pressure to eat was associated with modestly lower later BMIz. These feeding associations are likely bidirectional. They should not be read as causal. This was an observational, complete-case analysis of an unweighted public-use cohort. The findings describe associations within WIC-enrolled children. They may not generalize to other groups. Within these limits, the late preschool period (48–60 months) may be an important window for obesity prevention. WIC could be a useful platform for this work. Possible targets include responsive feeding, prenatal smoking cessation, and early nutrition education. Future studies should test whether WIC-based feeding interventions can shift BMIz trajectories and reduce obesity disparities.

## Figures and Tables

**Table 1:** Baseline characteristics of WIC-enrolled caregiver–child dyads (analytic sample, N = 2,247).

Domain/Characteristic	Mean ± SD or n (%)
Child BMI-for-age z-score (BMIz)	
BMIz (all waves pooled)	0.51 ± 1.29 (n = 5,583)
BMIz at 24 months	0.46 ± 1.25 (n = 998)
BMIz at 36 months	0.50 ± 1.27 (n = 1,503)
BMIz at 48 months	0.50 ± 1.34 (n = 1,654)
BMIz at 60 months	0.56 ± 1.29 (n = 1,428)
Baseline Characteristics	
Individual (Child-Level Factors)	
Gender Female Male	1098 (48.7%) 1149 (51.3%)
Ethnicity (n = 2,239) Not Hispanic or Latino Hispanic or Latino	1217 (54.4%) 1022 (45.6%)
Race (n = 2,223) White Black or African American All Other	1303 (58.6%) 529 (23.8%) 391 (17.6%)
Birth weight (kg)	3.23 ± 0.50
Birth Complications No Yes	1989 (88.5%) 258 (11.5%)
Interpersonal (Caregiver-Level Factors)	
Maternal age at child’s birth, years 16–19 20–25 26 or older	234 (10.4%) 915 (40.7%) 1098 (48.9%)
Marital Status at Baseline Not married (including divorced and widowed) Married	1546 (68.8%) 701 (31.2%)
Parity First born Second born Third or subsequent	924 (41.1%) 622 (27.7%) 701 (31.2%)
BMI Category of Mother at Screening Normal or Underweight Overweight Obesity	1025 (45.6%) 552 (24.6%) 670 (29.8%)
Prenatal smoking, cigarettes/day Did not smoke 1–9 cigarettes 10–20 cigarettes	2001 (89.3%) 177 (7.9%) 62 (2.8%)
Drinking Alcohol at Baseline Never Drink Alcohol Drink Alcohol	2214 (98.8%) 27 (1.2%)
High Education Level (n = 2240) High school or Less More than High school	1374 (61.3%) 866 (38.7%)
Paternal Presence in Household No Yes	1001 (44.6%) 1243 (55.4%)
Paternal Weight Category Too thin Normal weight Overweight	112 (5.0%) 1891 (85.1%) 220 (9.9%)
Postpartum Depressive Symptoms (EPDS) <10 ≥10 or higher	1897 (89.4%) 225 (10.6%)
Household Factors	
Food Security Score at Baseline High or Marginal Food Security Low Food Security Very Low Food Security	1149 (51.1%) 711 (31.6%) 387 (17.2%)
Participation in Other Benefit Programs at Baseline: No other benefit programs SNAP (alone or with other programs) Other programs (excluding SNAP)	355 (15.8%) 1107 (49.3%) 785 (34.9%)
Timing of WIC Enrollment 1st trimester 2nd trimester 3rd trimester Postnatal	753 (33.5%) 896 (39.9%) 323 (14.4%) 275 (12.2%)
Poverty Level at Baseline ≤75% of poverty guideline Above 75–130% of poverty guideline Above 130% of poverty guideline	1410 (62.8%) 613 (27.3%) 224 (10.0%)

Note. N = 2,247 unique children contributing 5,583 BMIz observations across four assessment waves. BMIz values at 24 months are restricted to CDC 2000 growth chart-derived observations (children measured at ≥24 months of age) to ensure a consistent reference standard across all waves. Overall BMIz reflects pooled observations across all four waves. Percentages may not sum to 100 due to rounding or variable-specific missingness; denominators for variables with missing data are indicated in parentheses. Prenatal alcohol use was not included in multivariable models due to very low prevalence (1.2%), which precluded stable parameter estimation.

**Table 2:** Time-varying caregiver feeding practices across early childhood.

	N; Mean ± SD		
Feeding Practice	15 Months	42 Months	54 Months
Pressure to eat	1,699; 2.97 ± 1.13	1,892; 3.18 ± 1.10	1,854; 3.23 ± 1.09
Restriction	1,698; 3.53 ± 1.37	1,891; 3.43 ± 1.39	1,854; 3.37 ± 1.37

Note. Values are presented as N; mean ± standard deviation. N reflects unique caregiver–child dyads in the analytic cohort with a valid feeding practice measurement at each time point, irrespective of BMI-for-age z-score (BMIz) availability. Feeding practices were assessed using caregiver-reported subscales adapted from the Comprehensive Feeding Practices Questionnaire (CFPQ) [24], scored on a 5-point Likert scale (1 = never, 5 = always); higher scores indicate greater use of each practice. Practices were modeled as lagged, time-varying predictors: 15-month measurements were used as predictors of BMIz at 24 and 36 months, 42-month measurements as predictors of BMIz at 48 months, and 54-month measurements as predictors of BMIz at 60 months. Sample sizes vary across time points due to participant attrition and item-level missingness.

**Table 3:** Unconditional mixed-effects model of child BMI-for-age z-score from 24 to 60 months.

	Estimate	SE	95% CI	*p*-Value
Intercept (24 months)	0.45	0.04	0.38, 0.51	<0.001
36 months	0.04	0.04	−0.03, 0.11	0.307
48 months	0.04	0.03	−0.03, 0.11	0.219
60 months	0.12	0.04	0.05, 0.19	0.001
**Random Effects (Variance)**				
Between-child variance	1.050	0.040	0.974, 1.132	-
Within-child variance (residual)	0.612	0.015	0.584, 0.642	-
ICC	0.632	0.011	0.609, 0.654	-

Note. Model estimated using restricted maximum likelihood (REML) with a random intercept for child. N = 5,583 observations from 2,247 children. The reference category for time is 24 months; coefficients represent mean differences in BMIz relative to 24 months. The intraclass correlation coefficient (ICC) reflects the proportion of total BMIz variance attributable to stable between-child differences.

**Table 4:** Fully adjusted mixed-effects model predictors of child BMI-for-age z-score from 24 to 60 months (significant predictors only).

Variable	β, 95% CI
Time (ref: 24 months) 48 months 60 months	0.10 * (0.016, 0.184) 0.18 *** (0.090, 0.261)
Individual (Child-Level Factors)	
Birth weight (kg)	0.51 *** (0.398, 0.623)
Male (vs. female)	−0.16 ** (−0.265, −0.055)
Hispanic or Latino (vs. non-Hispanic)	0.19 ** (0.069, 0.310)
Birth complication (yes vs. no)	0.25 ** (0.081, 0.424)
Interpersonal (Caregiver-Level Factors)	
Maternal age 20–25 (vs. 16–19)	−0.24 * (−0.435, −0.041)
Maternal weight status (ref: normal/underweight) Overweight Obesity	0.16 * (0.033, 0.295) 0.41 *** (0.282, 0.529)
Biological father weight category: Overweight (vs. underweight)	0.35 *, 0.06 to 0.65
Prenatal smoking (ref: non-smoker) 1–9 cigarettes/day 10–20 cigarettes/day	0.47 *** (0.266, 0.670) 0.49 ** (0.171, 0.817)
More than high school (vs ≤ HS)	−0.13 * (−0.248, −0.020)
Caregiver Feeding Practices (lagged, time-varying)	
Pressure to eat	−0.04 * (−0.076, −0.007)
Restriction	0.07 *** (0.039, 0.097)
Random Effects	
Between-child variance	0.88 (0.807, 0.968)
Within-child variance (residual)	0.64 (0.608, 0.678)
ICC	0.58 [0.551, 0.607]

Note. Model estimated using restricted maximum likelihood (REML) with a random intercept for child. N = 4,314 observations from 1,738 children. All covariates listed were included in the model; only statistically significant predictors are displayed in the main rows. Non-significant covariates retained in the model but not shown include child race, marital status, maternal age ≥ 26 years, poverty level, food security, participation in other benefit programs, and timing of WIC enrollment (all *p* > 0.05). Feeding practice coefficients reflect lagged associations: feeding practices measured at 15 months were used to predict BMI-for-age z-score (BMIz) at 24 and 36 months, feeding practices measured at 42 months were used to predict BMIz at 48 months, and feeding practices measured at 54 months were used to predict BMIz at 60 months. Reference time point is 24 months. ICC = intraclass correlation coefficient. * *p* < 0.05. ** *p* < 0.01. *** *p* < 0.001.

## Data Availability

Data described in the manuscript, the codebook, and analytic code are publicly available at: https://agdatacommons.nal.usda.gov/articles/dataset/WIC_Infant_and_Toddler_Feeding_Practices_Study-2_WIC_ITFPS-2_Prenatal_Infant_Year_5_Year_Datasets/24668343 (accessed on 15 October 2025).

## Supplementary Materials

The following supporting information can be downloaded at https://www.mdpi.com/article/10.3390/nu18142249/s1.

## Abbreviations

The following abbreviations are used in this manuscript:

BMI	Body Mass Index
BMIz	BMI-for-Age z-Score
WIC	Special Supplemental Nutrition Program for Women, Infants, and Children
SEM	Social Ecological Model
ITFPS-2	Infant and Toddler Feeding Practices Study-2
CFPQ	Comprehensive Feeding Practices Questionnaire
EPDS	Edinburgh Postnatal Depression Scale
SNAP	Supplemental Nutrition Assistance Program
FPL	Federal Poverty Level
USDA	U.S. Department of Agriculture
FNS	Food and Nutrition Service
CDC	Centers for Disease Control and Prevention
WHO	World Health Organization
ECHO	Environmental Influences on Child Health Outcomes
REML	Restricted Maximum Likelihood
ICC	Intraclass Correlation Coefficient
CI	Confidence Interval
SE	Standard Error
SD	Standard Deviation
STROBE	Strengthening the Reporting of Observational Studies in Epidemiology

## References

[1] Michael N., Gupta V., Fogel A., Huang J., Chen L., Sadananthan S.A., Ong Y.Y., Aris I.M., Pang W.W., Yuan W.L. (2023). Longitudinal characterization of determinants associated with obesogenic growth patterns in early childhood. Int. J. Epidemiol..

[2] Aris I.M., Rifas-Shiman S.L., Li L.J., Kleinman K.P., Coull B.A., Gold D.R., Hivert M.F., Kramer M.S., Oken E. (2019). Patterns of body mass index milestones in early life and cardiometabolic risk in early adolescence. Int. J. Epidemiol..

[3] Liu C., Chow S.M., Aris I.M., Dabelea D., Neiderhiser J.M., Leve L.D., Blair C., Catellier D.J., Couzens L., Braun J.M. (2025). Early-Life Factors and Body Mass Index Trajectories Among Children in the ECHO Cohort. JAMA Netw. Open.

[4] Rolland-Cachera M.F., Deheeger M., Maillot M., Bellisle F. (2006). Early adiposity rebound: Causes and consequences for obesity in children and adults. Int. J. Obes..

[5] Woo Baidal J.A., Locks L.M., Cheng E.R., Blake-Lamb T.L., Perkins M.E., Taveras E.M. (2016). Risk Factors for Childhood Obesity in the First 1000 Days: A Systematic Review. Am. J. Prev. Med..

[6] Arisaka O., Ichikawa G., Koyama S., Sairenchi T. (2020). Childhood obesity: Rapid weight gain in early childhood and subsequent cardiometabolic risk. Clin. Pediatr. Endocrinol..

[7] Chung S.T., Onuzuruike A.U., Magge S.N. (2018). Cardiometabolic risk in obese children. Ann. N. Y. Acad. Sci..

[8] Emmerich S.D., Ogden C.L. (2024). QuickStats: Prevalence of Obesity and Severe Obesity Among Persons Aged 2–19 Years—United States, 1999–2000 Through 2021–2023. Morb. Mortal. Wkly. Rep..

[9] Hu K., Staiano A.E. (2022). Trends in Obesity Prevalence Among Children and Adolescents Aged 2 to 19 Years in the US From 2011 to 2020. JAMA Pediatr..

[10] U.S. Department of Agriculture Food and Nutrition Service (2025). About WIC. https://www.fna.usda.gov/wic.

[11] Gundersen C., Kreider B., Pepper J.V. (2017). Partial identification methods for evaluating food assistance programs: A case study of the causal impact of SNAP on food insecurity. Am. J. Agric. Econ..

[12] Tester J.M., Rosas L.G., Leung C.W. (2020). Food Insecurity and Pediatric Obesity: A Double Whammy in the Era of COVID-19. Curr. Obes. Rep..

[13] Chaparro M.P., Anderson C.E. (2021). Differences in Early Childhood Dietary Behaviors by Infant Feeding Type and Sex. J. Nutr..

[14] Weinfield N.S., Anderson C.E. (2022). Postpartum Symptoms of Depression are Related to Infant Feeding Practices in a National WIC Sample. J. Nutr. Educ. Behav..

[15] Rossi A., Chen Z.H., Ahmadiankalati M., Campisi S.C., Reyna M.E., Dempsey K., Jenkins D., O’Connor D., El-Sohemy A., Mandhane P.J. (2025). Determining the interplay of prenatal parental BMI in shaping child BMI trajectories: The CHILD Cohort Study. Int. J. Obes..

[16] Oudat Q., Miller E.L., Couch S.C., Lee R.C., Bakas T. (2025). Understanding Caregivers’ Influence on Preschoolers’ Eating Behaviors: An Integrative Review Guided by the Theory of Planned Behavior. Children.

[17] Bronfenbrenner U. (1977). Toward an experimental ecology of human development. Am. Psychol..

[18] Borger C., Zimmerman T., DeMatteis J., Gollapudi B., Whaley S., Ritchie L., Au L., May L. (2022). WIC Infant and Toddler Feeding Practices Study-2: Fifth Year Report. Food and Nutrition Service, US Department of Agriculture. https://fns-prod.azureedge.us/sites/default/files/resource-files/WIC-ITFPS2-Year5Report.pdf.

[19] USDA FNS Office of Policy Support (2022). WIC Infant and Toddler Feeding Practices Study-2 (WIC ITFPS-2): Prenatal, Infant Year 5 Year Datasets. https://agdatacommons.nal.usda.gov/articles/dataset/WIC_Infant_and_Toddler_Feeding_Practices_Study-2_WIC_ITFPS-2_Prenatal_Infant_Year_5_Year_Datasets/24668343.

[20] Von Elm E., Altman D.G., Egger M., Pocock S.J., Gøtzsche P.C., Vandenbroucke J.P. (2007). The Strengthening the Reporting of Observational Studies in Epidemiology (STROBE) statement: Guidelines for reporting observational studies. Bull. World Health Organ..

[21] Harrison G.G., Hirschman J.D., Owens T.A., McNutt S.W., Sallack L.E. (2014). WIC Infant and Toddler Feeding Practices Study: Protocol design and implementation. Am. J. Clin. Nutr..

[22] Kuczmarski R.J. CDC Growth Charts: United States. 2000: US Department of Health and Human Services, Centers for Disease Control and Prevention (CDC). https://www.cdc.gov/nchs/data/series/sr_11/sr11_246.pdf.

[23] World Health Organization (WHO) (2006). WHO Child Growth Standards: Length/Height-for-Age, Weight-for-Age, Weight-for-Length, Weight-for-Height and Body Mass Index-for-Age: Methods and Development. https://www.who.int/publications/i/item/924154693X.

[24] Musher-Eizenman D., Holub S. (2007). Comprehensive Feeding Practices Questionnaire: Validation of a new measure of parental feeding practices. J. Pediatr. Psychol..

[25] StataCorp LLC (2025). Stata Statistical Software, version 18.

[26] Mattsson M., Murray D.M., Hawkes C.P., Kiely M., Ní Chaoimh C., McCarthy F.P., Biesma R., Boland F. (2021). Body Mass Index Trajectories in the First 5 Years and Associated Antenatal Factors. Front. Pediatr..

[27] Reyna M.E., Petersen C., Dai D.L.Y., Dai R., Becker A.B., Azad M.B., Miliku K., Lefebvre D.L., Moraes T.J., Mandhane P.J. (2022). Longitudinal body mass index trajectories at preschool age: Children with rapid growth have differential composition of the gut microbiota in the first year of life. Int. J. Obes..

[28] De Craemer M., Cardon G., Decraene M., Androutsos O., Moreno L., Iotova V., Koletzko B., Socha P., Manios Y., Verbestel V. (2024). Longitudinal changes in preschoolers’ adiposity indicators according to compliance with 24-hour movement behavior guidelines: Results from the ToyBox-study. BMC Public Health.

[29] Kapral N., Miller S.E., Scharf R.J., Gurka M.J., DeBoer M.D. (2018). Associations between birthweight and overweight and obesity in school-age children. Pediatr. Obes..

[30] Qiao Y., Ma J., Wang Y., Li W., Katzmarzyk P.T., Chaput J.P., Fogelholm M., Johnson W.D., Kuriyan R., Kurpad A. (2015). Birth weight childhood obesity: A12-country study. Int. J. Obes. Suppl..

[31] Schellong K., Schulz S., Harder T., Plagemann A. (2012). Birth weight and long-term overweight risk: Systematic review and a meta-analysis including 643,902 persons from 66 studies and 26 countries globally. PLoS ONE.

[32] Yu Z.B., Han S.P., Zhu G.Z., Zhu C., Wang X.J., Cao X.G., Guo X.R. (2011). Birth weight and subsequent risk of obesity: A systematic review and meta-analysis. Obes. Rev..

[33] Jiang Y., Hu J., Chen F., Liu B., Wei M., Xia W., Yan Y., Xie J., Du S., Tian X. (2025). Comprehensive systematic review and meta-analysis of risk factors for childhood obesity in China and future intervention strategies. Lancet Reg. Health West. Pac..

[34] Thurber K.A., Dobbins T., Kirk M., Dance P., Banwell C. (2015). Early life predictors of increased body mass index among indigenous Australian children. PLoS ONE.

[35] Diesel J.C., Eckhardt C.L., Day N.L., Brooks M.M., Arslanian S.A., Bodnar L.M. (2015). Gestational Weight Gain and Offspring Longitudinal Growth in Early Life. Ann. Nutr. Metab..

[36] Crespi C.M., Gao S., Payne A., Nobari T.Z., Avila A., Nau C., Whaley S.E., Wang M.C. (2021). Longitudinal trajectories of adiposity-related measures from age 2-5 years in a population of low-income Hispanic children. Pediatr. Res..

[37] Mayer A., Herr R.M., Klein T., Wiedemann E., Diehl K., Hoffmann S., Blume M., Jepsen D., Sundmacher L., Andreas M. (2023). Socio-economic inequalities in body mass index among preschool children: Do sports programs in early childhood education and care centers make a difference?. Front. Public Health.

[38] El-Gamal F.M., Babader R., Al-Shaikh M., Al-Harbi A., Al-Kaf J., Al-Kaf W. (2020). Study determinants of increased Z-Score of Body Mass Index in preschool-age children. BMC Res. Notes.

[39] Ogden C.L., Carroll M.D., Lawman H.G., Fryar C.D., Kruszon-Moran D., Kit B.K., Flegal K.M. (2016). Trends in Obesity Prevalence Among Children and Adolescents in the United States, 1988–1994 Through 2013–2014. JAMA.

[40] Skinner A.C., Ravanbakht S.N., Skelton J.A., Perrin E.M., Armstrong S.C. (2018). Prevalence of Obesity and Severe Obesity in US Children, 1999–2016. Pediatrics.

[41] Guerrero A.D., Mao C., Fuller B., Bridges M., Franke T., Kuo A.A. (2016). Racial and Ethnic Disparities in Early Childhood Obesity: Growth Trajectories in Body Mass Index. J. Racial Ethn. Health Disparities.

[42] Lucas J.A., Marino M., Bailey S.R., Espinoza K., Datta R., Boston D., Heintzman J. (2025). Trends in Pediatric Obesity Prevalence Among Community Health Center Patients by Latino Ethnicity and Nativity, 2012–2020. Ann. Fam. Med..

[43] Bekelman T.A., Dabelea D., Ganiban J.M., Law A., McGovern Reilly A., Althoff K.N., Mueller N., Camargo CAJr Duarte C.S., Dunlop A.L., Elliott A.J. (2021). Regional and sociodemographic differences in average BMI among US children in the ECHO program. Obesity.

[44] Ayebare E., Hanson C., Nankunda J., Hjelmstedt A., Nantanda R., Jonas W., Tumwine J.K., Ndeezi G. (2022). Factors associated with birth asphyxia among term singleton births at two referral hospitals in Northern Uganda: A cross sectional study. BMC Pregnancy Childbirth.

[45] Ernstmeyer K., Christman E. (2025). Healthy Newborn Care. Nursing Health Promotion.

[46] Zivaljevic J., Jovandaric M.Z., Babic S., Raus M. (2024). Complications of Preterm Birth-The Importance of Care for the Outcome: A Narrative Review. Medicina.

[47] Council N.R. (2009). Weight Gain During Pregnancy: Reexamining the Guidelines.

[48] Barroso C.S., Roncancio A., Hinojosa M.B., Reifsnider E. (2012). The association between early childhood overweight and maternal factors. Child. Obes..

[49] Heslehurst N., Vieira R., Akhter Z., Bailey H., Slack E., Ngongalah L., Pemu A., Rankin J. (2019). The association between maternal body mass index and child obesity: A systematic review and meta-analysis. PLoS Med..

[50] Deveci A.C., Keown-Stoneman C.D.G., Maguire J.L., O’Connor D.L., Anderson L.N., Dennis C.L., Birken C.S. (2023). TARGet Kids! Collaboration. Maternal BMI in the preconception period, and association with child zBMI growth rates. Pediatr. Obes..

[51] Lu Z., Shadid I.L.C., Shah J., Carey V.J., Laranjo N., O’Connor G.T., Zeiger R.S., Bacharier L., Litonjua A.A., Weiss S.T. (2025). Impact of maternal body mass index (BMI) and gestational weight gain on offspring’s weight and BMI z-scores across the first 8 years of life. Clin. Obes..

[52] Mei H., Guo S., Lu H., Pan Y., Mei W., Zhang B., Zhang J. (2018). Impact of parental weight status on children’s body mass index in early life: Evidence from a Chinese cohort. BMJ Open.

[53] Dereń K., Wyszyńska J., Nyankovskyy S., Nyankovska O., Łuszczki E., Sobolewski M., Mazur A. (2020). Assessment of the Impact of Parental BMI on the Incidence of Overweight and Obesity in Children from Ukraine. J. Clin. Med..

[54] Perkins J., Re T., Ong S., Niu Z., Wen X. (2023). Meta-Analysis on Associations of Timing of Maternal Smoking Cessation Before and During Pregnancy with Childhood Overweight and Obesity. Nicotine Tob. Res..

[55] Oken E., Levitan E.B., Gillman M.W. (2008). Maternal smoking during pregnancy and child overweight: Systematic review and meta-analysis. Int. J. Obes..

[56] Albers L., von Kries R., Sobotzki C., Gao H.J., Buka S.L., Clifton V.L., Grzeskowiak L.E., Oken E., Paus T., Pausova Z. (2018). Differences in maternal smoking across successive pregnancies—Dose-dependent relation to BMI z-score in the offspring: An individual patient data (IPD) meta-analysis. Obes. Rev..

[57] Møller S.E., Ajslev T.A., Andersen C.S., Dalgård C., Sørensen T.I.A. (2014). Risk of childhood overweight after exposure to tobacco smoking in prenatal and early postnatal life. PLoS ONE.

[58] da Silva Magalhães E.I., Peixoto Lima N., Baptista Menezes A.M., Gonçalves H., Wehrmeister F.C., Formoso Assunção M., Horta B.L. (2019). Maternal smoking during pregnancy and offspring body composition in adulthood: Results from two birth cohort studies. BMJ Open.

[59] Mekonnen T., Brantsæter A.L., Andersen L.F., Lien N., Arah O.A., Gebremariam M.K., Papadopoulou E. (2022). Mediators of differences by parental education in weight-related outcomes in childhood and adolescence in Norway. Sci. Rep..

[60] Hsu P.C., Hwang F.M., Chien M.I., Mui W.C., Lai J.M. (2022). The impact of maternal influences on childhood obesity. Sci. Rep..

[61] Lee H., Keller K.L. (2012). Children who are pressured to eat at home consume fewer high-fat foods in laboratory test meals. J. Acad. Nutr. Diet..

[62] Shloim N., Edelson L.R., Martin N., Hetherington M.M. (2015). Parenting Styles, Feeding Styles, Feeding Practices, and Weight Status in 4–12 Year-Old Children: A Systematic Review of the Literature. Front. Psychol..

[63] Balantekin K.N., Andrade A.L.P., Ziegler A.M., Temple J.L. (2024). Restriction and Pressure to Eat Are Associated Cross-Sectionally, But Not Longitudinally, With BMI z-Score in a Longitudinal Cohort Study of Adolescents. Child. Obes..

[64] Quah P.L., Ng J.C., Fries L.R., Chan M.J., Aris I.M., Lee Y.S., Yap F., Godfrey K.M., Chong Y.S., Shek L.P. (2019). Longitudinal Analysis Between Maternal Feeding Practices and Body Mass Index (BMI): A Study in Asian Singaporean Preschoolers. Front. Nutr..

[65] Fisher J.O., Eicher-Miller H.A., Odoms-Young A., Palacios C., Abrams S., Andres A., Byrd-Bredbenner C., Deierlein A., Lawless M., Momin S. (2024). Parental and Caregiver Feeding Styles and Practices and Growth, Body Composition and Risk of Obesity: A Systematic Review.

[66] Kac G., Schlüssel M.M., Pérez-Escamilla R., Velásquez-Melendez G., da Silva A.A. (2012). Household food insecurity is not associated with BMI for age or weight for height among Brazilian children aged 0-60 months. PLoS ONE.

[67] Wirth S.H., Palakshappa D., Brown C.L. (2020). Association of household food insecurity and childhood weight status in a low-income population. Clin. Obes..

[68] Jones-Smith J.C., Dieckmann M.G., Gottlieb L., Chow J., Fernald L.C.H. (2014). Socioeconomic status and trajectory of overweight from birth to mid-childhood: The Early Childhood Longitudinal Study-Birth Cohort. PLoS ONE.

[69] Metallinos-Katsaras E., Must A., Gorman K. (2012). A longitudinal study of food insecurity on obesity in preschool children. J. Acad. Nutr. Diet..

[70] Woo Baidal J.A., Nelson C.C., Perkins M., Colchamiro R., Leung-Strle P., Kwass J.A., Gortmaker S.L., Davison K.K., Taveras E.M. (2017). Childhood obesity prevention in the women, infants, and children program: Outcomes of the MA-CORD study. Obesity.

