# Supplementary material for: Caregiver Feeding Practices and Late-Preschool BMI-for-Age z-Score Trajectories Among WIC-Enrolled Children: A National Longitudinal Study
Source: Nutrients. 2026 Jul 9;18(14):2249. doi: 10.3390/nu18142249 (PMC13414580; doi:10.3390/nu18142249)
Supplement: Supplementary file 1 [file nutrients-18-02249-s001.zip › nutrients-4375296-supplementary.pdf]

Supplement Materials

Supplementary Note S1. WIC Program Eligibility and ITFPS-2 Study Inclusion Criteria

WIC Program Eligibility Criteria

The Special Supplemental Nutrition Program for Women, Infants, and Children (WIC) is a federally funded nutrition assistance program administered by the U.S. Department of Agriculture Food and Nutrition Service (USDA FNS). To be eligible for WIC, individuals must meet all of the following criteria:

- Categorical eligibility: Must be a pregnant woman, postpartum woman (up to 6 months after delivery or end of pregnancy), breastfeeding woman (up to 12 months postpartum), infant (up to 12 months of age), or child (ages 1 through 4 years, up to the 5th birthday).
- Residency: Must reside in the state in which they apply.
- Income: Household income must be at or below 185% of the U.S. Federal Poverty Level (FPL), or the applicant must participate in Medicaid, the Supplemental Nutrition Assistance Program (SNAP), or Temporary Assistance for Needy Families (TANF), which confers automatic income eligibility.
- Nutritional risk: Must be certified as being at nutritional risk by a health professional. Nutritional risk may be determined through anthropometric measures (e.g., low birth weight, underweight, overweight, short stature), biochemical or hematological indicators (e.g., anemia), dietary patterns associated with inadequate or inappropriate nutrient intake, or medical conditions that predispose individuals to nutritional deficiency or inadequate growth.

WIC provides supplemental nutritious foods, nutrition education and counseling, breastfeeding promotion and support, and referrals to health and social services. Additional program details are available at https://www.fns.usda.gov/wic.

ITFPS-2 Study Inclusion Criteria

The WIC Infant and Toddler Feeding Practices Study-2 (ITFPS-2) enrolled caregiver–infant dyads from WIC agencies across the United States between July 2013 and January 2014. Study-specific eligibility criteria included:

- Infant age ≤ 3 months at WIC enrollment
- Primary caregiver (typically the mother) able to complete interviews in English or Spanish
- Dyad not enrolled exclusively in a supplemental WIC sample (supplemental samples were collected to oversample specific subpopulations but are excluded from the public-use longitudinal file)

Data were collected through structured caregiver interviews conducted at multiple time points from early infancy through the child's fifth birthday. Standardized anthropometric measurements (child weight and length/height) were obtained from WIC administrative records. Detailed descriptions of sampling, weighting, and data collection procedures have been published previously (Borger et al., 2022; Harrison et al., 2014; U.S. Department of Agriculture Food and Nutrition Service, 2025).

Supplementary Table S1. Sensitivity analysis: fully adjusted mixed-effects model excluding birthweight

Variable	Primary Model (M4_Full) β (SE), 95% CI	Sensitivity Model (No Birthweight) β (SE), 95% CI
**Time (ref: 24 months)**		
36 months	0.08 (0.04), −0.00 to 0.17	0.09* (0.04), 0.00 to 0.18
48 months	0.10* (0.04), 0.02 to 0.18	0.11* (0.04), 0.02 to 0.19
60 months	0.18*** (0.04), 0.09 to 0.26	0.18*** (0.04), 0.09 to 0.27
**Caregiver Feeding Practices**		
Pressure to eat — lagged	−0.04* (0.02), −0.08 to −0.01	−0.04* (0.02), −0.08 to −0.01
Restriction — lagged	0.07*** (0.01), 0.04 to 0.10	0.07*** (0.01), 0.04 to 0.10
**Individual (Child-Level)**		
Birthweight (kg)	0.51*** (0.06), 0.40 to 0.62	—
Male (vs. female)	−0.16** (0.05), −0.27 to −0.06	−0.08 (0.05), −0.19 to 0.02
Black or African American (vs. White)	0.08 (0.07), −0.06 to 0.23	0.01 (0.08), −0.14 to 0.16
All other race (vs. White)	−0.03 (0.07), −0.17 to 0.12	−0.02 (0.08), −0.16 to 0.13
Hispanic or Latino (vs. non-Hispanic)	0.19** (0.06), 0.07 to 0.31	0.18** (0.06), 0.06 to 0.31
Birth complication (yes vs. no)	0.25** (0.09), 0.08 to 0.42	0.00 (0.08), −0.16 to 0.17
**Interpersonal (Caregiver-Level)**		
Maternal age 20–25 years (vs. 16–19)	−0.24* (0.10), −0.44 to −0.04	−0.21* (0.10), −0.42 to −0.01
Maternal age ≥26 years (vs. 16–19)	−0.16 (0.11), −0.38 to 0.05	−0.12 (0.11), −0.34 to 0.10

Maternal married (vs. not married)	−0.10 (0.07), −0.25 to 0.04	−0.10 (0.07), −0.25 to 0.04
Second born (vs. first born)	0.05 (0.07), −0.08 to 0.19	0.05 (0.07), −0.09 to 0.19
Third or subsequent born (vs. first born)	−0.03 (0.08), −0.18 to 0.12	−0.01 (0.08), −0.16 to 0.14
Maternal overweight (vs. normal weight)	0.16* (0.07), 0.03 to 0.30	0.19** (0.07), 0.05 to 0.32
Maternal obesity (vs. normal weight)	0.41*** (0.06), 0.28 to 0.53	0.46*** (0.06), 0.33 to 0.58
Smoking 1–9 cig/day (vs. none)	0.47*** (0.10), 0.27 to 0.67	0.42*** (0.11), 0.22 to 0.63
Smoking 10–20 cig/day (vs. none)	0.49** (0.16), 0.17 to 0.82	0.43* (0.17), 0.10 to 0.76
Education > high school (vs. ≤ high school)	−0.13* (0.06), −0.25 to −0.02	−0.12* (0.06), −0.24 to −0.01
Baseline: Father in household: yes (vs. no)	0.04 (0.06), −0.09 to 0.16	0.06 (0.07), −0.07 to 0.19
Biological father weight category: normal weight (vs. underweight)	0.12 (0.13), −0.13 to 0.37	0.15 (0.13), −0.11 to 0.41
Biological father weight category: overweight (vs. underweight)	0.35* (0.15), 0.06 to 0.65	0.38* (0.15), 0.08 to 0.68
Edinburgh Postpartum Depression Scale ≥10 (vs. <10)	−0.03 (0.09), −0.20 to 0.14	−0.04 (0.09), −0.22 to 0.13
**Household (Contextual-Level)**		
Low food security (vs. high/marginal)	0.01 (0.06), −0.10 to 0.13	0.04 (0.06), −0.08 to 0.16
Very low food security (vs. high/marginal)	0.01 (0.08), −0.14 to 0.16	0.01 (0.08), −0.14 to 0.17

Participates in SNAP or SNAP+other (vs. none)	−0.13 (0.08), −0.29 to 0.04	−0.13 (0.09), −0.30 to 0.04
Participates in other programs excluding SNAP (vs. none)	−0.01 (0.08), −0.17 to 0.15	−0.01 (0.08), −0.17 to 0.15
WIC 2nd trimester (vs. 1st trimester)	0.03 (0.06), −0.09 to 0.15	0.03 (0.06), −0.09 to 0.15
WIC 3rd trimester (vs. 1st trimester)	0.05 (0.08), −0.11 to 0.21	0.06 (0.08), −0.10 to 0.23
WIC postnatal (vs. 1st trimester)	−0.02 (0.10), −0.21 to 0.18	−0.03 (0.10), −0.23 to 0.17
Above 75%–130% of poverty guideline (vs. ≤75%)	−0.01 (0.06), −0.13 to 0.12	0.02 (0.06), −0.11 to 0.14
Above 130% of poverty guideline (vs. ≤75%)	−0.01 (0.09), −0.20 to 0.17	0.00 (0.10), −0.18 to 0.19
**Random Effects**		
Between-child variance	0.88 (0.04), 0.81 to 0.97	0.94 (0.04), 0.86 to 1.03
Within-child variance	0.64 (0.02), 0.61 to 0.68	0.64 (0.02), 0.61 to 0.68
Intraclass correlation (ICC)	0.58, 0.55 to 0.61	0.59, 0.57 to 0.62
**N observations**	4,314	4,314
**N children**	1,738	1,738

*Note. Both models are mixed-effects linear regressions with a random intercept for child (ITFPS2_ID), estimated using restricted maximum likelihood (REML). The primary model (M4_Full) corresponds to the fully adjusted model reported in Table 4 (N = 4,314 observations from 1,738 children). The sensitivity model excludes birthweight but retains all other covariates. The primary model column reports β, standard errors, and 95% confidence intervals; "—" indicates the variable was excluded from the model. *p < .05, **p < .01, ***p < .001.*

Reference

Borger, C., Zimmerman, T., DeMatteis, J., Gollapudi, B., Whaley, S., Ritchie, L., Au, L., & May, L. (2022). WIC Infant and Toddler Feeding Practices Study-2: Fifth Year Report. *Food and Nutrition Service, US Department of Agriculture. https://fns-prod. azureedge. us/sites/default/files/resource-files/WIC-ITFPS2-Year5Report-Appendices. pdf*.

Harrison, G. G., Hirschman, J. D., Owens, T. A., McNutt, S. W., & Sallack, L. E. (2014). WIC Infant and Toddler Feeding Practices Study: protocol design and implementation. *The American journal of clinical nutrition*, *99*(3), 742S-746S. https://doi.org/10.3945/ajcn.113.073585

U.S. Department of Agriculture Food and Nutrition Service. (2025). *About WIC*. Retrieved 05 November from https://www.fns.usda.gov/wic/about-wic
